# Lightweight deep learning system for automated bone age assessment in Chinese children: enhancing clinical efficiency and diagnostic accuracy

**DOI:** 10.3389/fendo.2025.1604133

**Published:** 2025-07-18

**Authors:** Pang Hai, Zhang Bin, Liu Kesheng, Li Cong, Xu Fei

**Affiliations:** Artificial Intelligence Research Center, Facilitate Healthy Developments for Children (Hebei) Technology Co., Ltd., Shijiazhuang, Hebei, China

**Keywords:** CH05, bone age assessment, lightweight deep neural network, YOLOv8, EfficientNetB3

## Abstract

Bone age assessment (BAA) is a critical diagnostic tool for evaluating skeletal maturity and monitoring growth disorders. Traditional clinical methods, however, are highly subjective, time-consuming, and reliant on clinician expertise, leading to inefficiencies and variability in accuracy. To address these limitations, this study introduces a novel lightweight two-stage deep learning framework based on the Chinese 05 BAA standard. In the first stage, the YOLOv8 algorithm precisely localizes 13 key epiphyses in hand radiographs, achieving a mean Average Precision (mAP) of 99.5% at Intersection over Union (IoU) = 0.5 and 94.0% within IoU 0.5–0.95, demonstrating robust detection performance. The second stage employs a modified EfficientNetB3 architecture for fine-grained epiphyseal grade classification, enhanced by the Rectified Adam (RAdam) optimizer and a composite loss function combining center loss and weighted cross-entropy to mitigate class imbalance. The model attains an average accuracy of 80.3% on the training set and 81.5% on the test set, with a total parameter count of 15.8 million—56–86% fewer than comparable models (e.g., ResNet50, InceptionV3). This lightweight design reduces computational complexity, enabling faster inference while maintaining diagnostic precision. This framework holds transformative potential for pediatric endocrinology and orthopedics by standardizing BAA, improving diagnostic equity, and optimizing resource use. Success hinges on addressing technical, ethical, and adoption challenges through collaborative efforts among developers, clinicians, and regulators. Future directions might include multimodal AI integrating clinical data (e.g., height, genetics) for holistic growth assessments.

## 1 Introduction

In medical practice, age evaluation encompasses two distinct measures: chronological age, defined as time elapsed since birth, and biological age, inferred from physiological markers such as skeletal maturity (1). Bone age, a critical subset of biological age, serves as a cornerstone for assessing developmental status from infancy through adolescence (2, 3). It correlates with growth velocity, pubertal onset, muscle mass, and bone density (4), and offering clinical utility in diagnosing growth disorders, monitoring therapeutic interventions (5), forensic applications (6), and athletic talent identification (7).

Bone age is predominantly evaluated via left-hand X-rays due to the anatomical richness of hand bones and standardized imaging protocols (8). The preference for the left hand stems from reduced injury prevalence in right-handed populations and adherence to early anthropometric conventions (9, 10). Since Greulich and Pyle’s seminal 1959 atlas (GP method) (11), which compares patient X-rays to standardized references, methodologies have evolved to include the Tanner-Whitehouse (TW) scoring system (TW2, TW3) (12, 13) and region-specific adaptations like the Chinese 05 standard (14). These techniques, however, remain labor-intensive and subjective, relying on clinician expertise and visual pattern recognition, which introduces variability in accuracy and diagnostic consistency, i.e., diabetic retinopathy (15) skin cancer (16), cataracts (17) and lung CT abnormalities (18–20).

In China, systematic bone age research emerged in the mid-20th century, with scholars like Liu Huifang and Zhang Naishu establishing early ossification benchmarks (21–23). Subsequent studies by Gu Guangning and Li Guozhen (24–26) laid the groundwork for localized standards, culminating in the CHN method (1992) (14), later revised as the Chinese 05 standard to reflect accelerated growth trends in children. Despite these advancements, manual assessment inefficiencies persist, exacerbated by rising clinical demands. With only 0.63 pediatricians per 1,000 Chinese children in 2019 [China Health Statistics Yearbook 2019], automating bone age evaluation is critical to alleviating physician workload and enhancing diagnostic throughput.

### 1.1 AI-driven solutions and recent advances

The integration of artificial intelligence (AI) into medical imaging has revolutionized diagnostics, as evidenced by applications in retinopathy screening and lung CT analysis (15–18). For bone age, deep learning models now address historical limitations. Early approaches, such as Jang et al. (27) regression-based CaffeNet model, achieved moderate accuracy (MAE: 6.4–18.9 months), while Hao et al. (28) carpal bone-focused CNN reduced errors to 2.75 months. Innovations like MobileNetV3-MLP hybrid (38) and GCN-CNN architectures mimicking clinical workflows (29, 30) further improved precision (MAE: 4.09–6.78 months). Notably, multicenter-validated AI system attained 84.6% accuracy within one year], and DCCGAN optimized both speed and accuracy over predecessors (31–33). These advancements underscore AI’s potential to standardize assessments, reduce subjectivity, and enable resource-efficient deployment across diverse healthcare settings (34, 35).

### 1.2 Proposed framework and clinical implications

**Figure 1:**
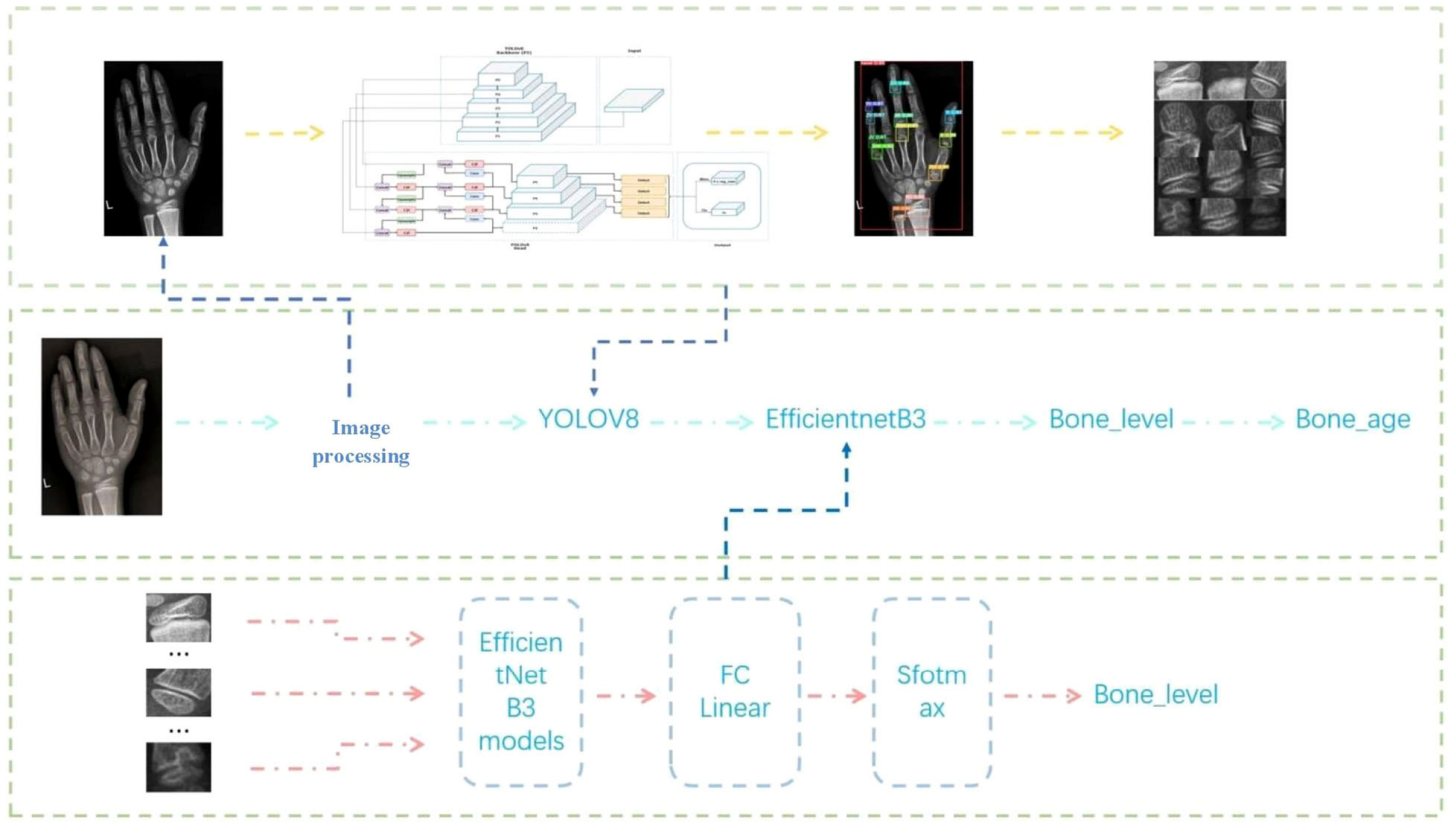
Flowchart of bone age recognition system.

Building on these foundations, we propose a lightweight two-stage model aligned with the Chinese 05 standard. Stage one localizes epiphyseal regions, while stage two classifies developmental features, enabling efficient integration with reference atlases (36). This architecture minimizes computational complexity, facilitating deployment in resource-constrained environments without sacrificing accuracy (37–39). By streamlining workflows and democratizing access, such systems promise to enhance diagnostic consistency, reduce costs, and expand clinical reach, ultimately bridging gaps in pediatric and endocrine care (40) (**Figure 1**). This evolution from manual atlases to AI-driven automation reflects a paradigm shift in bone age assessment, addressing longstanding challenges while paving the way for scalable, equitable healthcare solutions (41).

The primary objectives of this research are structured to address key challenges in automated bone age assessment through methodological innovation, robust data handling, and optimized model training. These objectives are outlined as follows:

### 1.3 Development of a lightweight two-stage bone age assessment model

Leveraging the “Chinese 05” standard, we propose a computationally efficient framework that decomposes bone age recognition into two stages:

**Stage 1 (Localization):** Utilize YOLOv8 (19) to detect and extract 13 clinically critical epiphyseal regions from hand X-ray images, prioritizing inference speed and precision.

**Stage 2 (Developmental Grading):** Implement a fine-grained EfficientNet-B3 (20) classifier to determine the developmental stage of each epiphysis, aligning with the “Chinese 05” scoring system.

The lightweight design is achieved through architectural optimizations, including channel pruning and quantization, to reduce computational complexity while maintaining diagnostic accuracy. Bone age is computed by aggregating developmental scores from all 13 regions, ensuring adherence to clinical standards.

### 1.4 Comprehensive data augmentation and preprocessing strategies

#### 1.4.1 YOLOv8 and EfficientNet model

- A foundational dataset of 3,182 high-quality X-ray images, manually annotated by 10 radiologists, is expanded 4× (to 12,728 images) using geometric transformations (rotation, flipping, cropping) and image stitching to improve spatial robustness.
- Preprocessing steps include grayscale conversion to reduce redundancy, contrast-limited adaptive histogram equalization (CLAHE) to enhance epiphyseal boundaries, and mean filtering to suppress noise, ensuring optimal feature extraction.
- A multicenter dataset of 10,608 images (from 100 hospitals) is augmented 4× (to 42,432 images) using identical geometric transformations to ensure consistency. Additional normalization and central cropping are applied to standardize inputs, minimizing domain shift across institutions.

**YOLOv8 Enhancements:** Integrate adaptive learning rate scheduling (Cosine Annealing) with the SGD optimizer to escape local minima and accelerate convergence. Adopt deterministic training (fixed seeds, controlled parallelism) to ensure reproducibility and reduce variance in detection performance.

**EfficientNet Enhancements:** Employ the RAdam (21) optimizer to stabilize training with dynamic variance rectification, coupled with a composite loss function:

**Weighted Cross-Entropy:** Address class imbalance by assigning higher weights to underrepresented developmental stages.

**Center Loss:** Improve feature discrimination by clustering embeddings of the same class, enhancing grading accuracy. Input preprocessing includes bilinear interpolation (to 384×384 resolution) and channel-wise normalization to align with pretrained weights.

## 2 Materials and methods

### 2.1 Dataset processing

This study leverages data from the bone age assessment system developed by Tongban Youkang Technology Co., Ltd., Hebei, China, company specializing in child health management ecosystems. Their integrated platform spans medical and household settings, offering services across health promotion, medical care, nutrition, medication, and insurance, with over 2,000 medical institutions served nationwide and approximately 3 million annual pediatric growth assessment reports. The research employs two core datasets:

**YOLOv8 Metacarpal and Phalangeal Bone Detection Dataset**: Contains 3,182 original X-ray images of metacarpal bones, annotated by 10 senior radiologists. Expanded to 12,728 images through data augmentation (4x increase).

**High-Quality Bone Age X-ray Dataset**: Comprises 10,608 images sourced from 100 hospitals (5,306 male, 5,302 female). Augmented to 42,432 images (4x increase), ensuring broad representation of bone ages (0–18 years).

**Ethical Compliance**: All images underwent anonymization to remove personal/patient identifiers, adhering strictly to medical data ethics. Data usage is restricted to bone age research to advance pediatric growth science.

**Preprocessing and Optimization:** To address variability in X-ray quality (e.g., lighting, angles, equipment), the following steps were implemented:

Grayscale conversion to prioritize bone morphology (epiphysis, diaphysis, growth plate) over color data.

**Figure 2:**
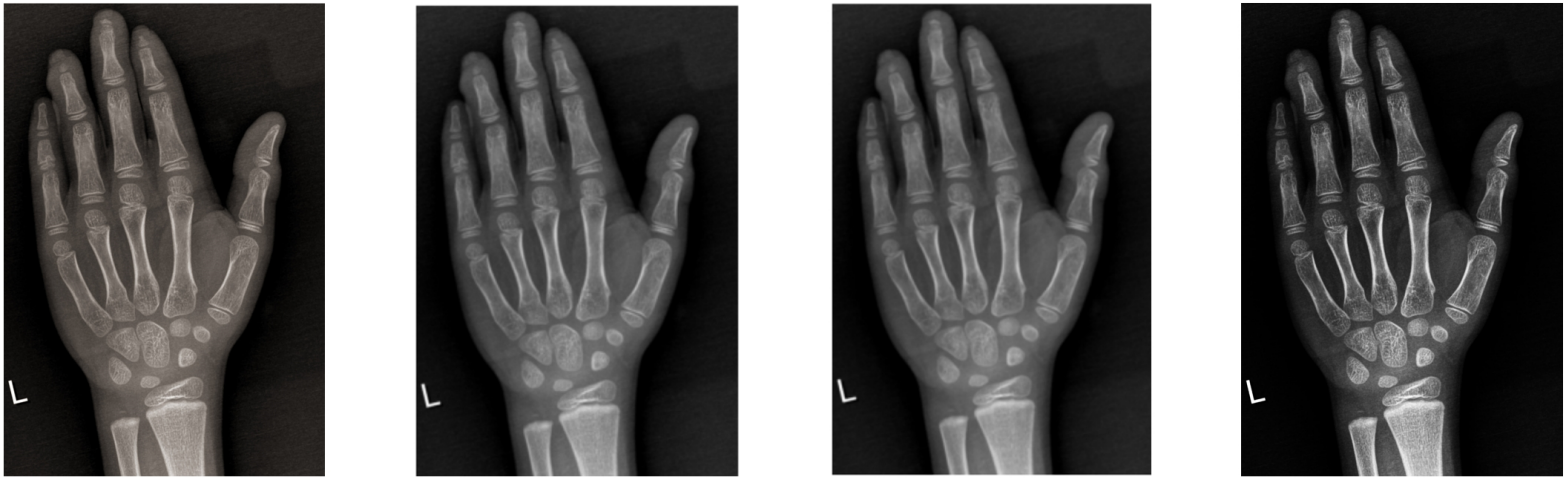
Image data processing.

Noise reduction via median filtering and contrast enhancement using histogram adjustments (**Figure 2**).

**Figure 3:**
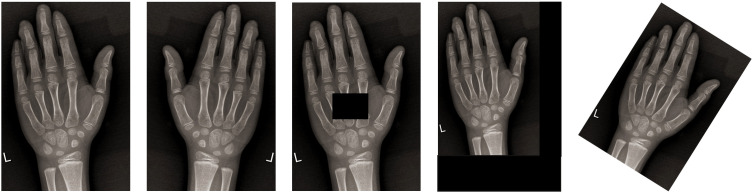
Image data augmentation.

Augmentation strategies (translation, cropping, rotation, flipping) to diversify training samples (**Figure 3**).

**Annotation Protocol:** Using LabelMe, 10 senior radiologists annotated 14 anatomical landmarks:

- *Radius, ulna, first/third/fifth metacarpals.*
- *First/third/fifth proximal phalanges, third/fifth middle phalanges, first/third/fifth distal phalanges.*
- *Entire hand region.*

**Figure 4:**
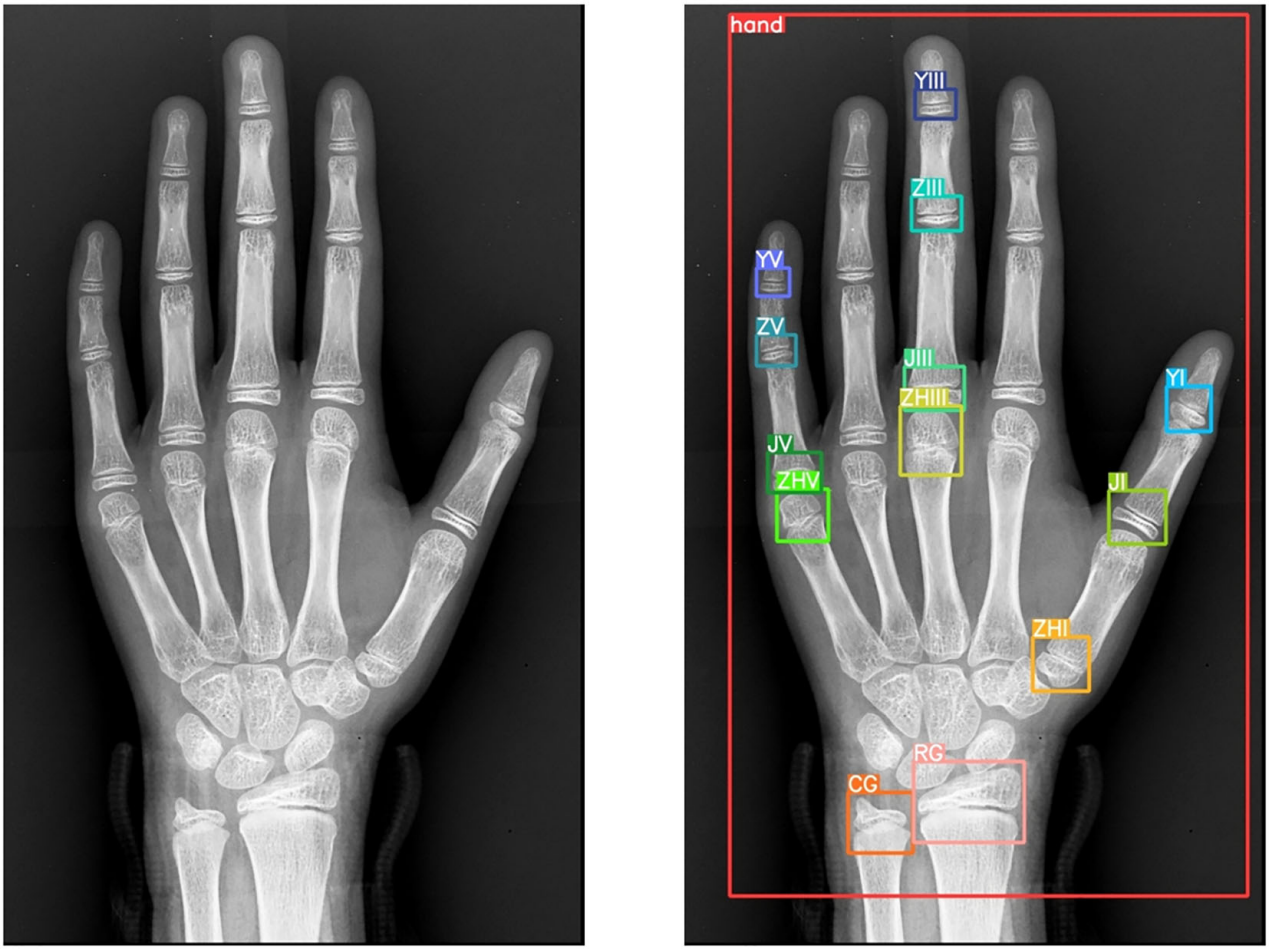
Data and annotation results.

This meticulous annotation process (**Figure 4**) ensured precision and reliability for model training.

#### 2.1.1 Data distribution and validation

**Figure 5:**
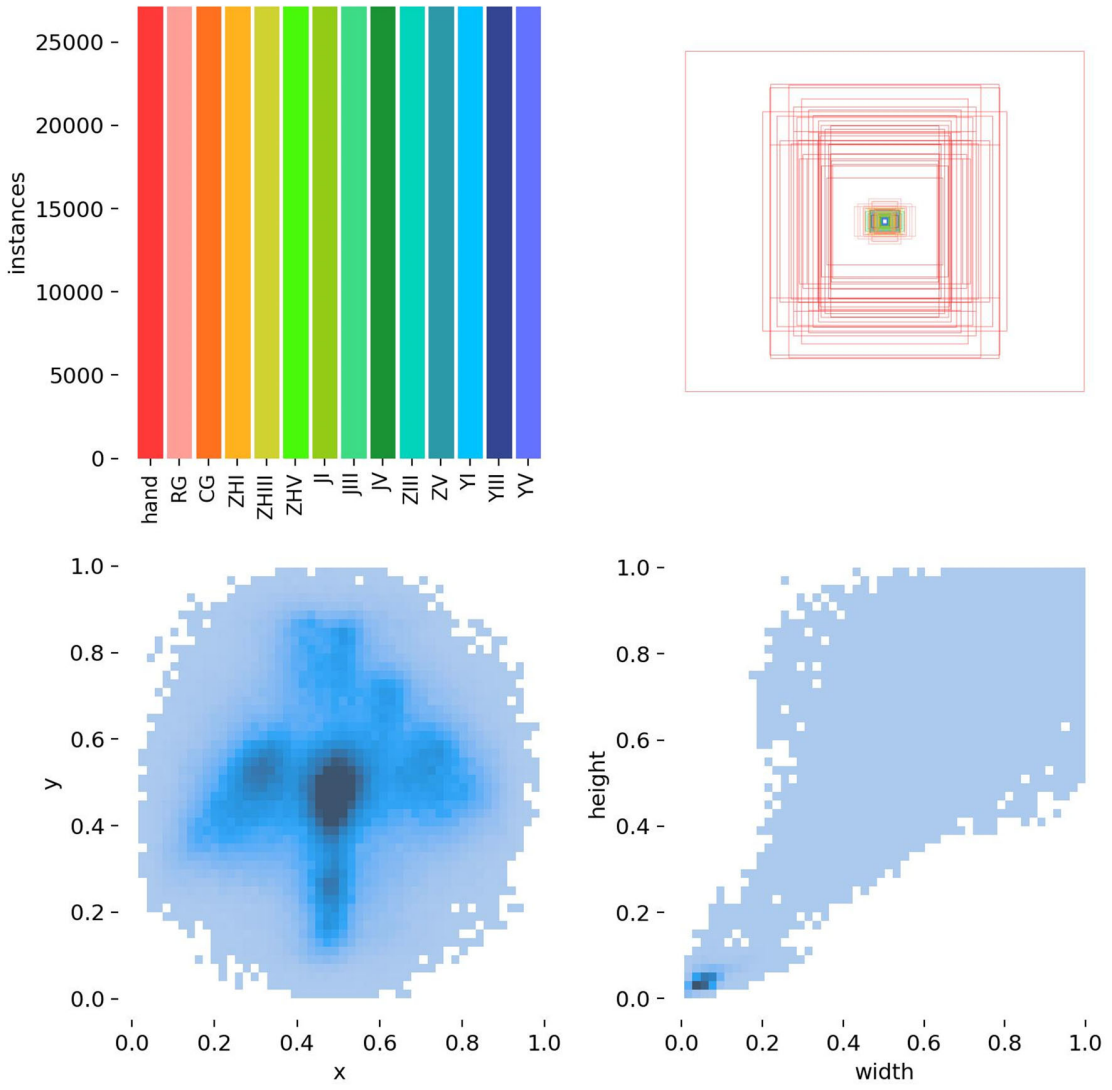
Distribution of object detection data.

**Figure 6:**
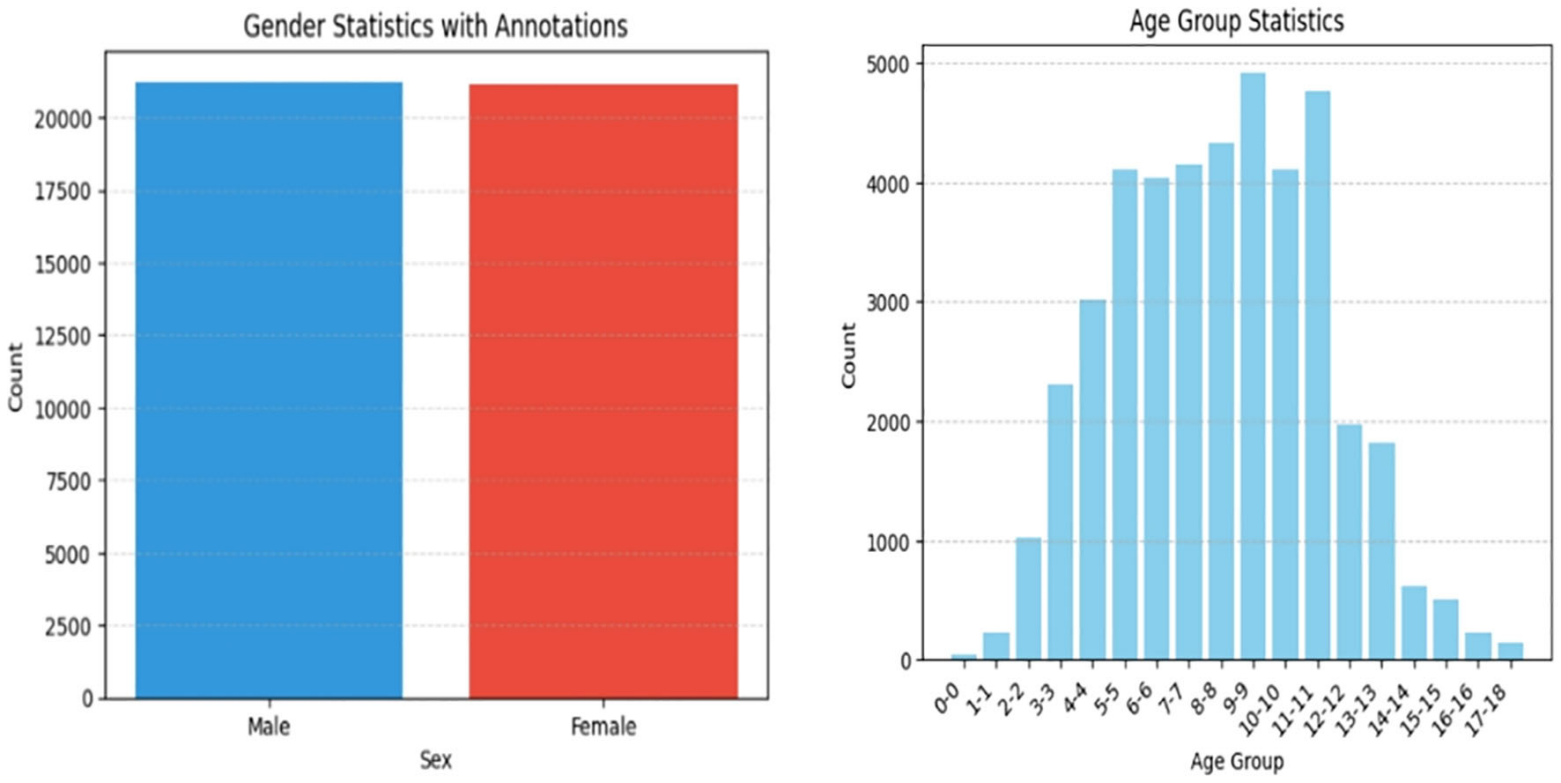
Data distribution for efficientnet classification.

**Figures 5** and **6** represents target detection data distribution and epiphyseal grade classifications and age demographics, confirming dataset diversity and research generalizability.

By integrating rigorous preprocessing, ethical safeguards, and expert annotations, this methodology establishes a robust foundation for advancing automated bone age assessment systems.

### 2.2 Research methods

This study adheres to the specifications of the Chinese Standard for Assessment of Skeletal Maturity and Prediction of Adult Height for Chinese Children and Adolescents (TY/T 3001-2006, hereafter “Chinese Standard 05”). Innovatively, the bone age recognition process is decomposed into two sequential, logically structured stages.

**Stage 1: Epiphyseal Region Extraction**

The initial stage focuses on precise extraction of key epiphyseal regions from wrist X-rays. The epiphysis, a critical indicator of skeletal development, provides essential insights into growth status and bone age assessment. To achieve this, the advanced YOLOv8 object detection model was employed. Leveraging its superior real-time detection capabilities and high precision, YOLOv8 efficiently localizes target epiphyseal regions within complex medical images, ensuring robust groundwork for subsequent analysis (19).

**Stage 2: Epiphyseal Grade Classification**

In the second stage, extracted epiphyseal regions are classified into distinct developmental grades based on the morphological criteria outlined in Chinese Standard 05. This classification demands both high accuracy and sensitivity to subtle morphological variations across developmental stages. The EfficientNet convolutional neural network (CNN) was selected for this task due to its optimized architecture and parameter efficiency, which enable high classification performance while maintaining computational economy (20). Post-classification, bone age values are calculated using the grading results and the computational framework prescribed by Chinese Standard 05. Comparative studies confirm the suitability and algorithmic superiority of YOLOv8 and EfficientNet in bone age recognition.

### 2.3 Model architectures

**Figure 7:**
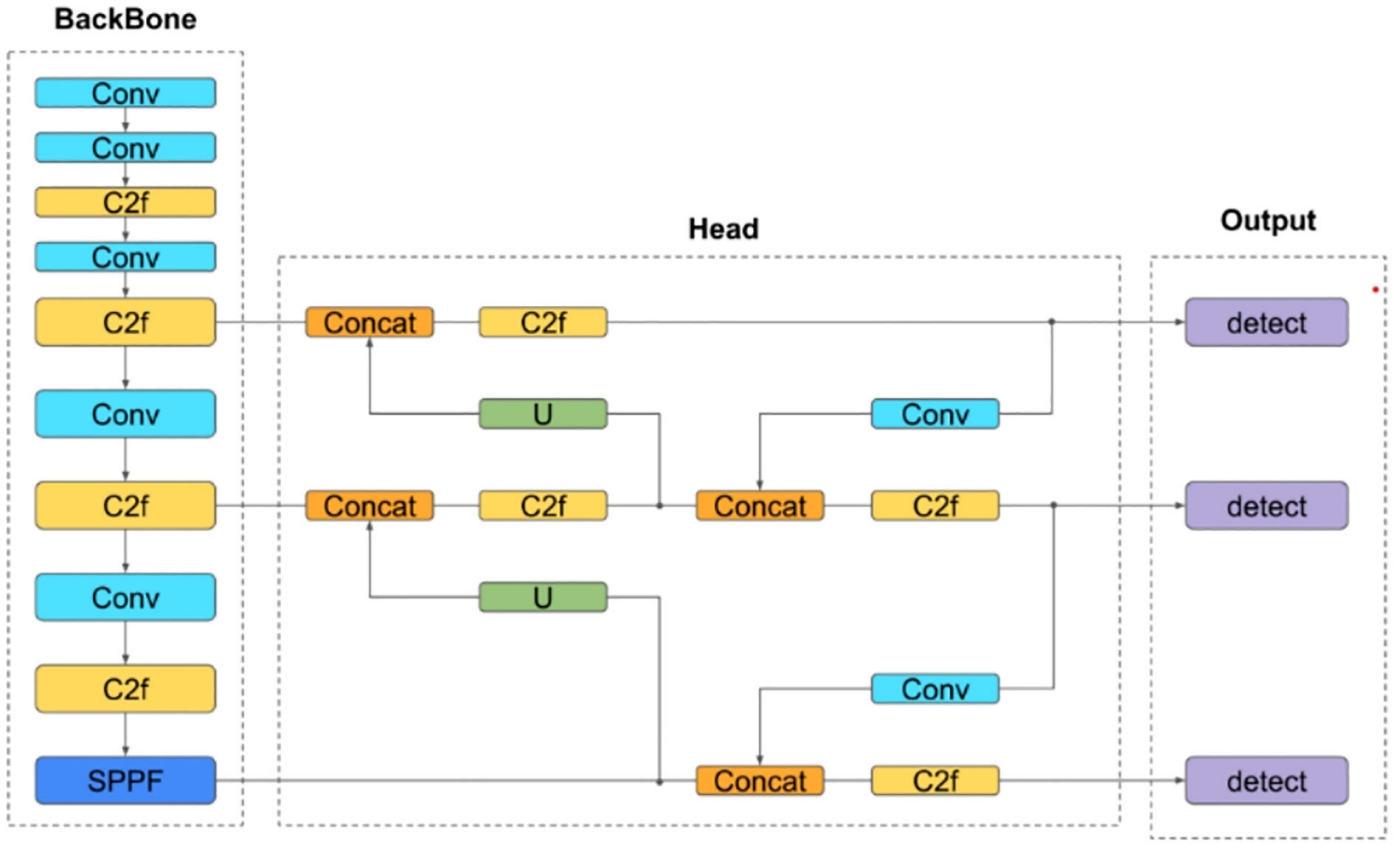
Backbone network of YOLOV8 model.

YOLOv8: As an enhanced iteration of YOLOv5, this one-stage detection model features improvements to its backbone network, detection head, and loss function. These refinements enable lightweight deployment across hardware platforms without compromising accuracy (**Figure 7**).

**Figure 8:**
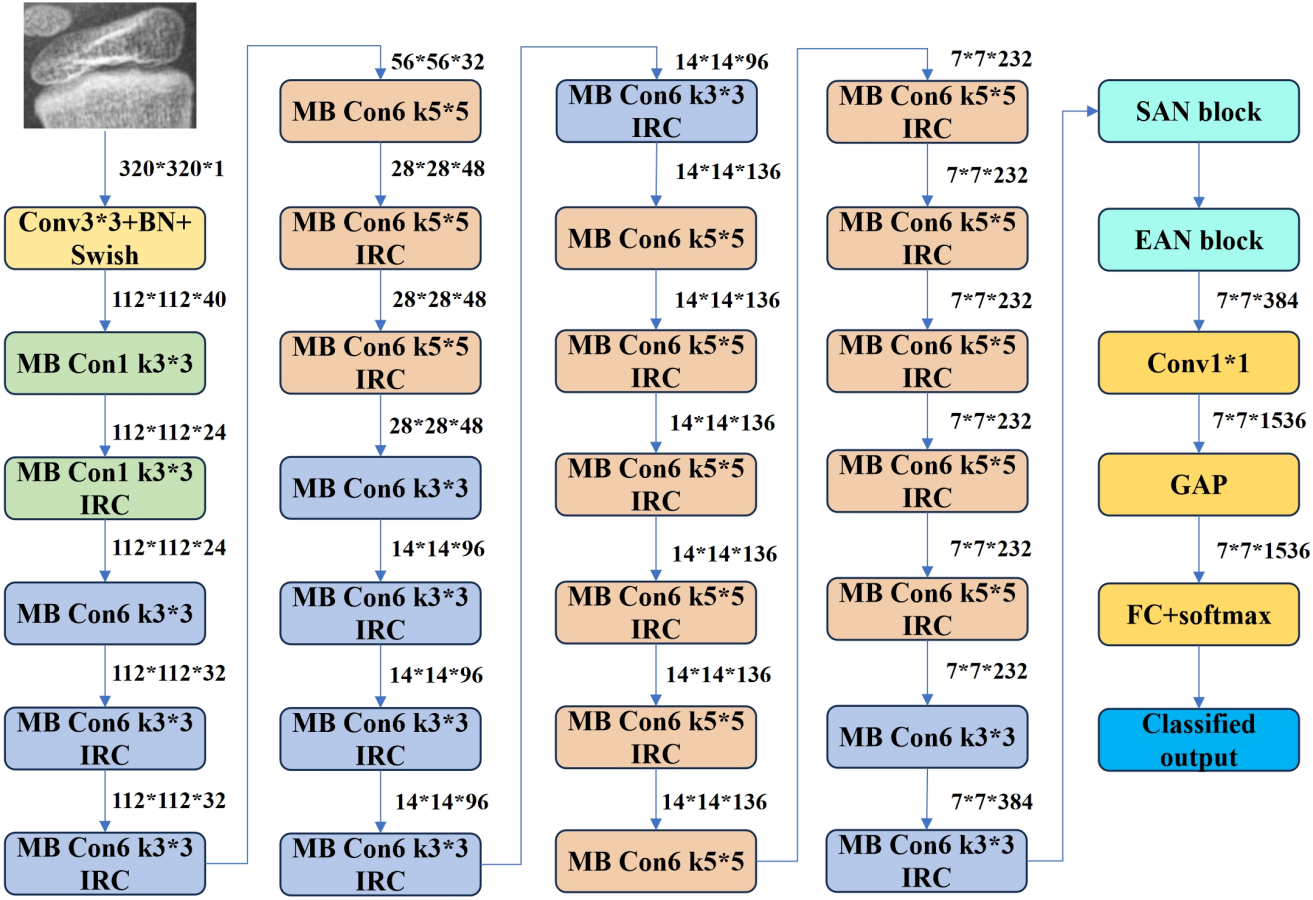
EfficientNet-B3 network structure.

EfficientNet B3: This CNN variant employs a compound scaling method to balance depth, width, and resolution for optimal efficiency. Its Mobile Inverted Bottleneck Convolution (MBConv) structure reduces computational overhead while preserving accuracy. Pre-trained on diverse datasets, EfficientNet B3 (**Figure 8**) was selected for its balance of performance and resource efficiency among the B0–B7 variants.

- Learning Rate: A dynamically adjusted learning rate (0.01 to 1e-5) was applied.
- Optimizer: Radam, an Adam variant with dynamic variance decay, was used to stabilize early-stage training.
- Center Loss: Penalizes deviations from class centroids using L2 norms, excelling in high-dimensional data but sensitive to outliers and computationally intensive with increasing classes.
- Weighted Cross-Entropy Loss: Addresses class imbalance by incorporating sample proportions as weights during parameter updates. Its convexity and differentiable nature facilitate gradient-based optimization while mitigating vanishing gradients.

Algorithmic details for the optimizer and loss functions are provided in **Tables 1** and **2**, respectively.

**Table 1:** Steps of the radam algorithm.

Radam Parameter Update Algorithm
Input: ${\left\{\alpha_{t}\right\}}_{t=1}^{T}$ : step size, $\left\{\beta_{1},\beta_{2}\right\}$ : decay rate to calculate moving average and moving 2^nd^ moment, $\theta_{0}$ : initial parameter, $f_{t}\left(\theta \right)$ : stochastic objective function. Output: $\theta_{t}$ : resulting parameters 1. Initialize moving 1^st^ and 2^nd^ moment $m_{0},v_{0}\leftarrow 0,0$ 2. Compute the maximum length of the approximated SMA $\rho_{\infty }=2/(1-\beta_{2})-1$ 3. While t={1,⋯,T} do $$g_{t}\leftarrow {\text{Δ}}_{\theta }f_{t}(\theta_{t-1})$$ $$v_{t}\leftarrow \beta_{2}v_{t-1}+(1-\beta_{2})g_{t}^{2}$$ $$m_{t}\leftarrow \beta_{1}m_{t-1}+(1-\beta_{1})g_{t}$$ $${\hat{m}}_{t}\leftarrow m_{t}/(1-\beta_{1}^{t})$$ $$\rho_{t}\leftarrow \rho_{\infty }-2t\beta_{2}^{t}/(1-\beta_{2}^{t})$$ If the variance is tractable, i.e., $\rho_{t}>4$ then $${\hat{v}}_{t}\leftarrow \sqrt{v_{t}/(1-\beta_{2}^{t})}$$ $$r_{t}\leftarrow \sqrt{\frac{(\rho_{t}-4)(\rho_{t}-2)\rho_{\infty }}{(\rho_{\infty }-4)(\rho_{\infty }-2)\rho t}}$$ $$\theta_{t}\leftarrow \theta_{t-1}-\alpha_{t}r_{t}{\hat{m}}_{t}/{\hat{v}}_{t}$$ Else $$\theta_{t}\leftarrow \theta_{t-1}-\alpha_{t}{\hat{m}}_{t}$$ Return $\theta_{T}$

**Table 2:** Steps of the loss function algorithm.

Calculation of Loss Function Algorithm
Input: Feature vector x , True labels $y_{true}$ , Predicted labels $y_{pre}$ , weight coefficients W . Output: Total loss L 1. Calculate the weight cross-entropy loss: $$L_{ce\_weighted}=-\sum_{c}^{C}w_{c}*y_{true}*\log(y_{pre})$$ 2. Calculate the center loss: a. Compute the Euclidean distance d between each sample’s feature vector x and the center of its corresponding class. b. Calculate the center loss. $$L_{center}=\frac{1}{2}*\sum_{i=1}^{N}*|x_{i}-c_{y_{i}}-{|}^{2}$$ Where N is the number of samples, $x_{i}$ is the feature vector of the i-th sample, and $c_{i}$ is the center of the class that the i-th sample belongs to. 3. Calculate the total loss L : $$L=L_{ce\_weighted}+\lambda L_{center}$$ Where λ is a hyperparameter that adjusts the weights between the two losses, and is set to 0.1 in this paper.

## 3 Results

### 3.1 Training the phalangeal and metacarpal epiphysis detection model using YOLOv8n

This study adopts a two-stage training approach, independently optimizing the object detection and classification models. The experimental setup (**Table 3**) utilizes the YOLOv8n architecture trained on hardware configured with a batch size of 256 and an initial learning rate of 0.01. The model underwent 500 epochs of training using the Adam optimizer, with a weight decay parameter of 0.001 to regularize learning. Transfer learning was applied by initializing the model with pre-trained YOLOv8n parameters, and an early stopping mechanism was integrated to prevent overfitting.

**Table 3:** Sever configuration.

Hardware	Specifications
Central Processing Unit (CPU)	Intel Core i9-14900kf
Graphics Card (GPU)	NVIDIA GeForce RTX 4090 24GB * 2 (Dual RTX 4090 with 24GB each)
Memory (RAM)	64GB
Storage	1TB M.2 NVMe Solid State Drive (SSD)

**Figure 9:**
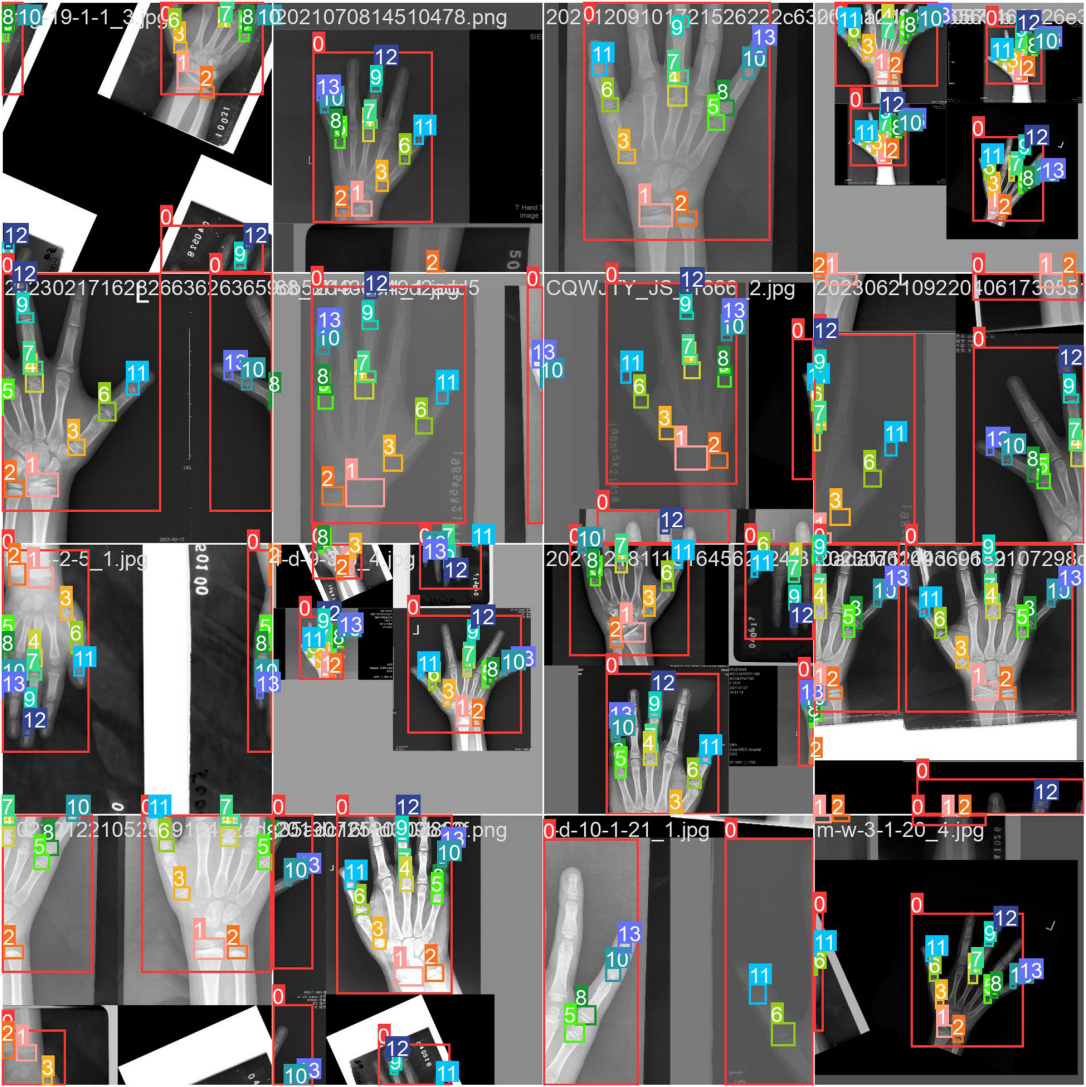
Mosaic and mixup augmentation.

The dataset comprised 8,910 training images and 3,818 test images. To bolster generalization, YOLOv8’s built-in data augmentation techniques were employed, including geometric transformations (flipping, rotation, cropping), photometric adjustments (brightness variation), and advanced strategies such as Mosaic and Mixup augmentation. As illustrated in **Figure 9**, the Mosaic method combines four distinct images into a single composite, dynamically varying object counts and positions to simulate diverse real-world scenarios. These augmentations collectively enhance the model’s robustness to input variability.

**Figure 10:**
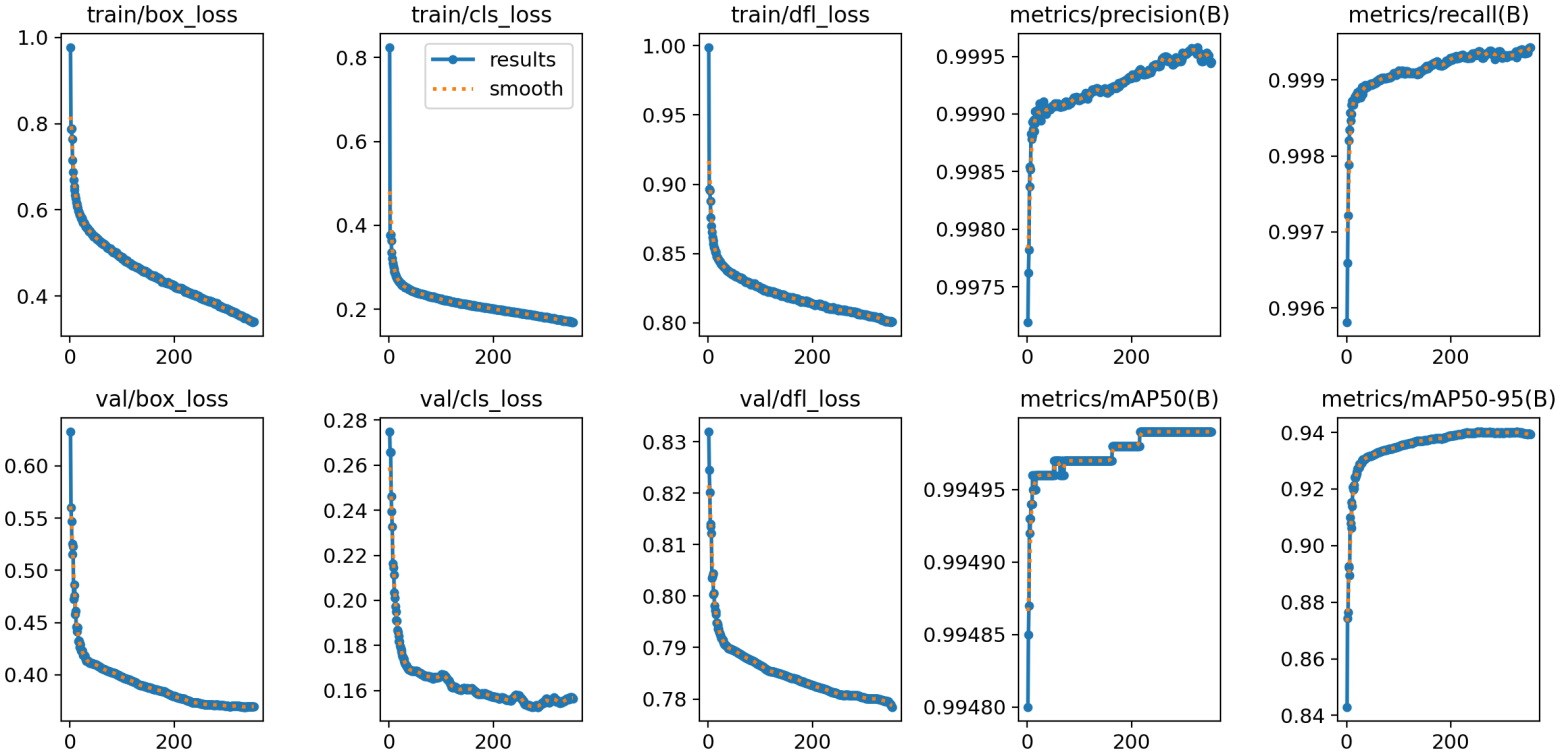
Training results.

**Figure 10** presents the training and validation outcomes of the YOLOv8 model. The box_loss, which quantifies the discrepancy between predicted and ground-truth bounding boxes, is computed based on Intersection over Union (IoU) values. Final box_loss values are 0.34 (training set) and 0.37 (test set). The cls_loss (classification loss), calculated via cross-entropy, assesses the accuracy of predicted object categories against their true labels, yielding 0.16 on the training set and 0.15 on the test set. Additionally, the dfl_loss (Distribution Focal Loss) enhances boundary localization accuracy by penalizing predictions with larger positional deviations between predicted and true bounding box centers. This loss registers 0.80 on the training set and 0.77 on the test set.

**Figure 11:**
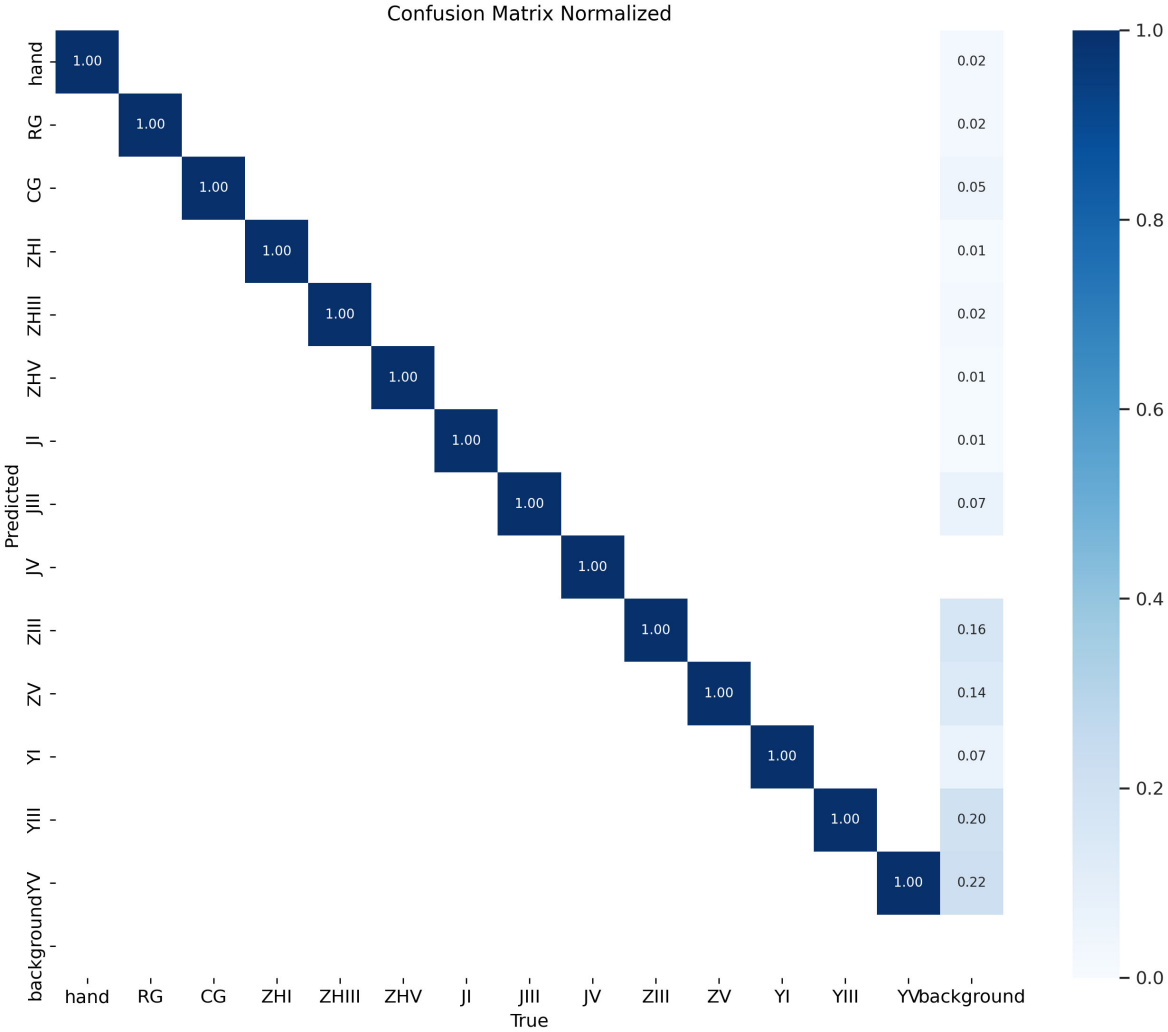
Confusion matrix for object detection.

The model demonstrates exceptional performance, achieving precision and recall rates of 99.95% at an Intersection over Union (IoU) threshold of 0.7. When evaluated under an IoU threshold of 0.5, it attains a mean Average Precision (mAP50) of 0.995, while the mAP50–95 score (spanning IoU thresholds from 0.5 to 0.95) reaches 0.939. As illustrated in **Figure 11**, the confusion matrix for the test set reveals near-perfect diagonal values, with detection accuracy for each epiphyseal location approaching 100%—highlighting the model’s outstanding recognition capabilities.

**Figure 12:**
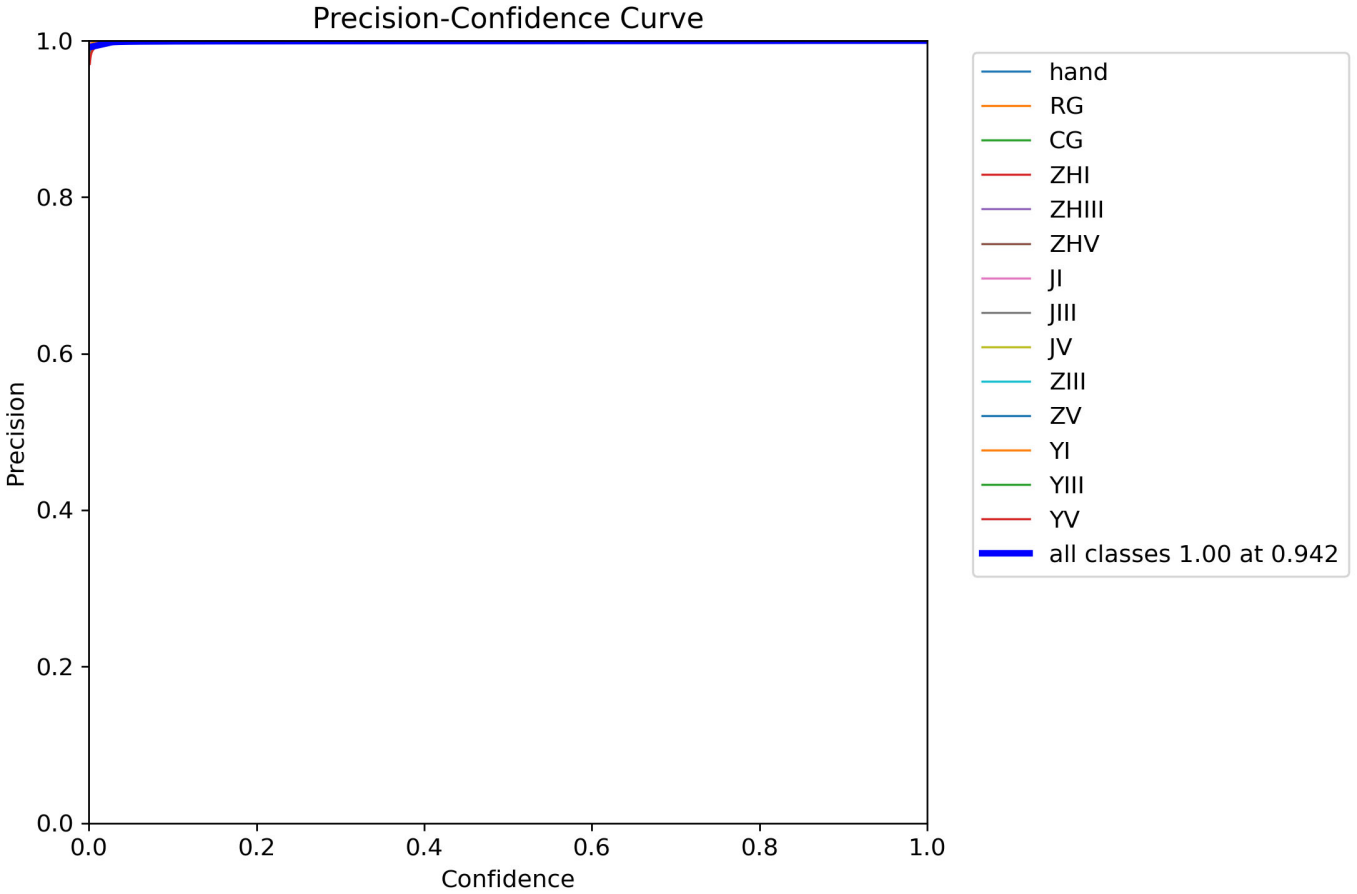
Model precision-confidence curve.

**Figure 13:**
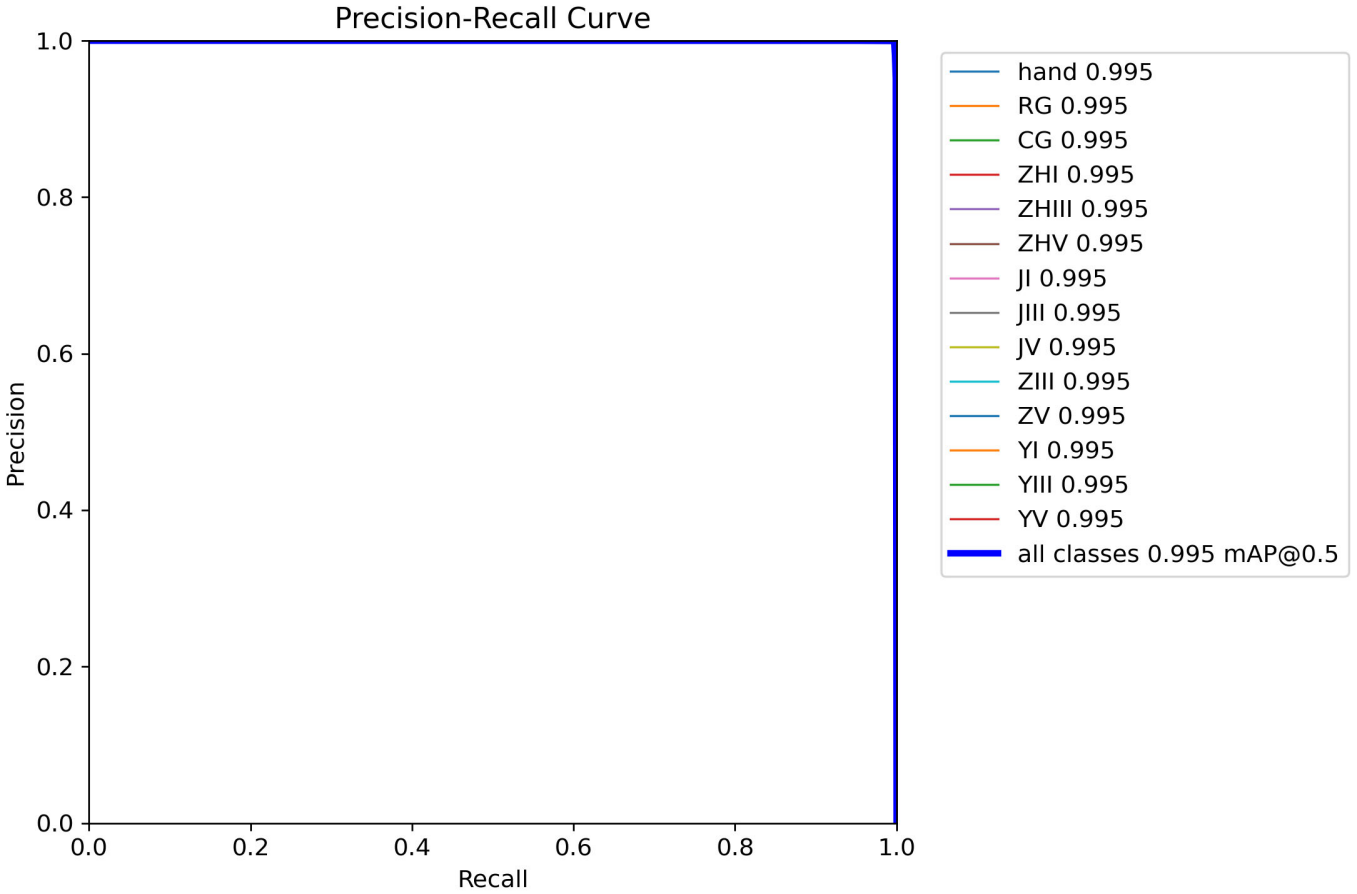
Precision-recall curve.

**Figure 12** demonstrates the precision-confidence curve of the model, showcasing how its prediction accuracy evolves as confidence levels change. This curve enables clinicians or researchers to assess the reliability of predictions at specific confidence thresholds, guiding decisions about when to trust the model’s outputs. **Figure 13** illustrates the Precision-Recall (PR) curve, which highlights the balance between precision (positive predictive value) and recall (sensitivity) across varying classification thresholds. By analyzing this curve, medical professionals gain insights into the model’s diagnostic performance under different operational conditions, such as its ability to minimize false positives or prioritize detecting true positives. Together, these visualizations offer actionable metrics to evaluate the model’s strengths and limitations, empowering healthcare providers to align its use with clinical priorities and improve patient care strategies.

As shown in **Table 4**, at an IoU threshold of 0.5, the YOLOv8 model outperforms other detectors with a mean average precision (mAP) of 0.995 on the test set. Comparatively, M2Det achieves an mAP of 0.785, Faster R-CNN attains 0.863, and YOLOv5 demonstrates a moderately higher but still suboptimal performance at 0.937. These results highlight YOLOv8’s superior accuracy in classifying phalangeal and metacarpal epiphyses (**Figure 13**).

**Table 4:** Comparison results of bone category classification among different models.

Model	mAP@0.5
Faster R-CNN	0.863
YOLOV5	0.937
M2Det	0.785
YOLOV8	0.995

### 3.2 Epiphyseal grade classification

**Figure 14:**
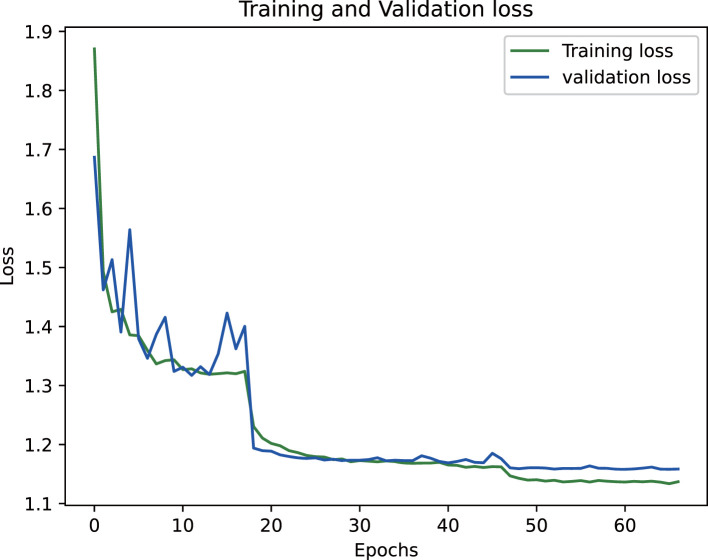
Training loss variation for epiphyseal grade classification.

**Figure 15:**
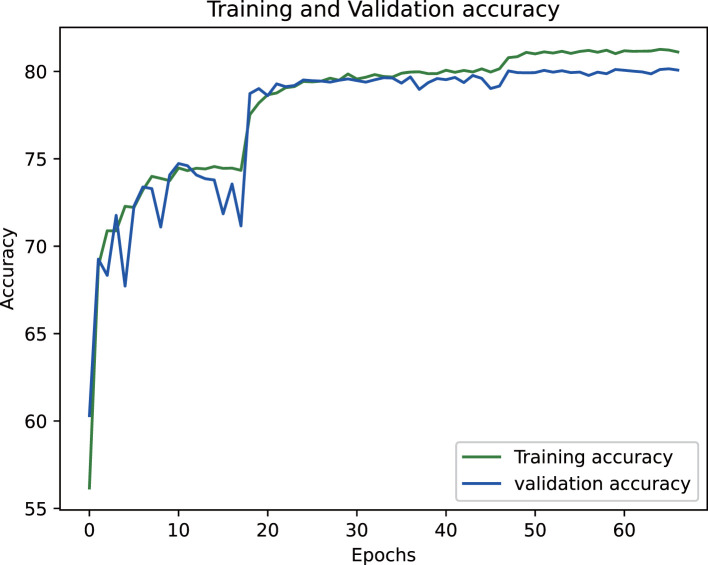
Training accuracy variation for epiphyseal grade classification.

The proposed epiphyseal grade classification model, built on the EfficientNetB3 architecture, achieved robust performance through optimized training strategies. The framework employs the Radam optimizer with adaptive learning rates to stabilize convergence and enhance generalization. A hybrid loss function combining weighted cross-entropy loss and center loss was implemented to simultaneously address class imbalance and improve feature discrimination by minimizing intra-class variations while maximizing inter-class differences. This dual-objective approach enabled the model to effectively capture nuanced distinctions between epiphyseal grades. Final evaluation yielded an accuracy of 81.5% on the training dataset and 80.3% on the test set, demonstrating strong consistency and minimal overfitting. The training dynamics, including the progressive reduction in loss values and convergence of accuracy metrics, are visualized in **Figures 14** and **15**, illustrating the model’s stable learning trajectory.

In the domain of bone age assessment, the proposed model in this study exhibits substantial advancements in predictive accuracy while simultaneously achieving notable progress in lightweight architecture and practical deployment. Evaluated on the RSNA dataset, our framework attains a mean absolute error (MAE) of 4.32 months—the lowest among existing methods—surpassing prior benchmarks by a significant margin. Comparatively, Iglovikov et al. (42) employed a two-stage approach, combining U-Net-based hand bone segmentation with a VGG regression network, yet achieved a higher MAE of 6.10 months. This performance gap likely stems from residual background noise and incomplete suppression of epiphyseal interference, which hindered feature learning efficiency. Bui et al. (43) adopted the TW3 assessment standard and utilized Faster R-CNN with InceptionV4 for region-of-interest analysis, but their methodology yielded a larger MAE of 7.08 months, indicating persistent challenges in minimizing systemic error. Similarly, integrated U-Net segmentation with Inception ResNet V2 training, reporting MAEs of 6.96 months (male), 7.35 months (female), and an overall average of 7.15 months. Despite achieving precise segmentation, their model’s structural complexity and extensive parameter count limited its practicality for clinical implementation (44).

Deshmukh et al. (45) employed FRCNN for key epiphyseal region detection, followed by training an RNN with an LSTM architecture, which yielded an average prediction error of 6.99 months. While their work introduced time-series modeling, the overall error rate remained suboptimal. In comparison, our framework leverages YOLOv8 for precise localization of the 13 key epiphyses specified in the Chinese 05 standard. This approach ensures accurate extraction of epiphyseal regions, eliminating background noise and irrelevant skeletal features that could obscure critical developmental signals. The improved localization enables more reliable identification of epiphyseal characteristics, directly addressing limitations in prior methodologies. Furthermore, we implemented a comprehensive suite of data augmentation techniques to suppress noise artifacts, enhancing the model’s robustness and significantly boosting its accuracy in bone age assessment.

Our model achieves competitive performance with just 15.8 million parameters, substantially fewer than existing models (51.04, 114.31, 35.84, and 69.81M), corresponding to parameter reductions of 69.04, 86.18, 55.92, and 77.31%, respectively. This streamlined architecture eliminates the need for expensive high-end hardware, making the model accessible to diverse medical facilities—including those lacking specialized computing infrastructure. Furthermore, under equivalent hardware conditions, our design enables faster computational speeds, improving clinical workflow efficiency and benefiting both healthcare providers and patients.

In summary, the proposed model not only attains state-of-the-art accuracy but also prioritizes practical deployability through its lightweight structure and optimized data processing. These advantages underscore its significant clinical value and broad applicability across resource-constrained settings. For detailed comparisons, refer to **Table 5**.

**Table 5:** Comparison results of different methods on the RSNA dataset.

Model	mAP@0.5	Parameters
U-Net+VGG-style+liner (42)	6.10	51.04M
Faster R-CNN + Inceptionv4 (43)	7.08	114.31M
U-Net+Inception-Resnet-V2 (44)	7.15	35.84M
Faster R-CNN + RNNS (45)	6.99	69.81M
YoloV8+EfficientNetb3	4.32	15.8M

To rigorously evaluate the model’s performance, this study utilized a dataset of 1,020 metacarpal and phalangeal X-ray images sourced from clinical practice, comprising electronic and scanned films as well as photographic reprints. This diverse collection represents real-world clinical scenarios, enabling a thorough assessment of the model’s robustness and accuracy across varying imaging conditions. On this validation set, the final model demonstrated strong performance, achieving a Top-3 accuracy of 99.04% and a Top-1 accuracy of 85.95%. Notably, the model attained 93.8% accuracy when predictions fell within 0.5 years of the actual bone age, underscoring its precision in age estimation.

The EfficientNet model achieved an average absolute age prediction error of 0.16 years on the 1,020-image dataset, demonstrating exceptional precision in bone age assessment. Remarkably, this performance was achieved with a parameter count of 15.8 million substantially fewer than comparative models—underscoring its streamlined architecture and computational efficiency. These lightweight properties position the model as a clinically practical solution, offering dual advantages, i.e., providing physicians with a reliable tool to enhance monitoring and management of pediatric growth and development, and reducing hardware cost demands, accelerating evaluation speed, and improving feasibility for widespread clinical adoption. Comprehensive performance metrics are detailed in **Tables 6** and **7**.

**Table 6:** Accuracy of the model for each epiphyseal stage on the 1020-image validation set.

Epiphyseal Regions	Top 3 Acc (%)	Top 1 Acc (%)
Radius	99.11	85.29
Ulna	98.72	83.63
Metacarpal I	99.51	87.84
Metacarpal III	98.72	87.16
Metacarpal V	99.41	86.27
Proximal Phalanx I	99.31	86.76
Proximal Phalanx III	99.71	87.45
Proximal Phalanx V	99.41	87.45
Middle Phalanx III	99.31	87.25
Middle Phalanx V	98.53	84.31
Distal Phalanx I	99.02	83.04
Distal Phalanx III	98.63	85.10
Distal Phalanx V	98.14	85.78

**Table 7:** Bone age recognition performance of different models.

Model	MAE (years)	Parameters
Resnet50	10.62	25.0M
InceptionV3	9.00	27.2M
Vgg16	8.53	43.28M
DenseNet161	7.62	28.7M
Inception Resnet V2	2.10	28.08M
EfficientNetb3	0.16	12.2M

## 4 Discussion

This paper introduces an innovative lightweight two-stage deep learning framework for bone age assessment, achieving marked improvements in accuracy and computational efficiency. In the first stage, the YOLOv8 model is employed for precise epiphyseal region-of-interest (ROI) detection. Aligned with the Chinese 05 bone age standard, the model accurately localizes and extracts 13 critical epiphyses from metacarpal X-ray images, establishing a robust foundation for subsequent analysis. The second stage performs fine-grained epiphyseal maturity grading using a customized EfficientNet-B3 architecture. This model is specifically trained to classify epiphyseal development stages according to the Chinese 05 grading criteria, ensuring clinically relevant evaluations (19, 20). By leveraging EfficientNet-B3’s lightweight design, the framework maintains high computational efficiency while minimizing resource demands, enhancing its practicality for real-world clinical deployment. Experimental results demonstrate that the proposed method significantly outperforms conventional approaches in both accuracy and processing speed, offering a scalable solution for automated bone age assessment systems (23, 25, 26).

To advance the performance and precision of the comprehensive assessment framework, systematic optimizations were implemented across both data processing and model training pipelines. These refinements not only enhanced the model’s robustness and generalization capabilities but also ensured consistent performance across diverse clinical settings (46). Through these targeted improvements, the lightweight two-stage bone age assessment framework presented in this study achieves state-of-the-art diagnostic accuracy while substantially improving operational efficiency. This dual focus on precision and resource optimization underscores the method’s clinical relevance, showcasing strong potential for widespread adoption in medical practice (33, 35, 46).

### 4.1 Dataset characteristics and optimization

This study leverages a multi-institutional dataset comprising over 10,000 metacarpal X-ray images, collected from more than 100 medical facilities across China. The dataset’s scale and diversity ensure broad representation of anatomical variations and clinical conditions. Annotation quality is enhanced by precise diagnostic labels derived from consensus interpretations by board-certified radiologists at participating institutions, ensuring reliability for training and validating high-precision recognition models (4, 8, 17, 47). To maximize data utility, a rigorous preprocessing pipeline was implemented, including standardized normalization for intensity variations, artifact reduction through adaptive filtering, and geometric augmentation techniques (e.g., rotation, flipping) to improve model generalizability. Spatial resolution alignment and region-of-interest cropping further refined input consistency. These steps collectively address heterogeneity inherent in multi-source medical imaging data while preserving diagnostically critical features (14, 30).

The image preprocessing pipeline began with grayscale conversion to eliminate interference between RGB color channels, concentrating image information on characteristic bone structure representation. The processed images then underwent geometric transformations - including rotation, translation, horizontal flipping, and random cropping - to artificially expand sample variation, thereby enhancing the model’s generalization capacity across diverse metacarpal X-ray variations (35, 43). To address inherent noise in medical imaging, a mean filtering operation was employed to reduce high-frequency interference while preserving critical anatomical features. This noise suppression strategy produced cleaner input data with optimized signal-to-noise ratios, simultaneously maintaining diagnostic relevance and improving feature discriminability. Collectively, these preprocessing stages established robust data-level foundations for developing high-performance recognition models by ensuring input standardization, augmenting pathological representation diversity, and enhancing feature extraction efficiency (45, 46).

### 4.2 Model optimization

This study proposes a composite lightweight deep learning framework specifically designed for bone age assessment. The architectural framework integrates two synergistic components: (1) the YOLOv8 object detection model, optimized to precisely localize and extract 13 epiphyseal regions critical to bone age evaluation as defined by the Chinese 05 standard; and (2) the EfficientNet-B3 classification network, fine-tuned to perform fine-grained classification of the detected epiphyseal regions according to the developmental stages outlined in the Chinese 05 standard. By combining YOLOv8’s high-precision localization capabilities with EfficientNet-B3’s parameter-efficient hierarchical feature learning, this hybrid architecture achieves robust performance while maintaining computational efficiency—a key requirement for clinical applications (6, 19, 20).

Throughout the training phase of the YOLOv8 object detection model, extensive data augmentation techniques—including image cropping, stitching, rotation, and geometric transformations—were implemented to enhance sample diversity and bolster the model’s generalization capabilities. To optimize parameter tuning, the Stochastic Gradient Descent (SGD) optimizer was employed in conjunction with an adaptive learning rate adjustment strategy, enabling systematic convergence during training (12, 15, 48). Furthermore, deterministic training configurations were adopted to minimize stochastic variability, ensuring consistent reproducibility and stable training outcomes.

### 4.3 Input processing

The EfficientNet-B3 framework implemented a standardized preprocessing sequence for image inputs. Initial resizing to 320×320 pixels was performed using bilinear interpolation, balancing computational efficiency with geometric preservation (8). Subsequent center-cropping to 300×300 pixels systematically removed peripheral noise while maintaining critical visual features. Pixel values were then normalized to the [-1, 1] range through linear scaling (x’ = x/127.5 - 1), a crucial transformation that stabilizes gradient magnitudes and accelerates model convergence.

Model optimization employed the Rectified Adam (RAdam) algorithm, which mitigates variance in parameter updates during early training phases. The learning objective combined two synergistic components:

**Center Loss**: Enhanced feature discriminability by minimizing intra-class variations while maximizing inter-class separation through class centroid alignment.

**Weighted Cross-Entropy**: Addressed class imbalance by incorporating frequency-adjusted weights during probability distribution alignment, ensuring robust performance across minority categories.

This dual-loss strategy simultaneously optimized categorical prediction accuracy and feature space organization, with gradient computations automatically balanced between loss components through backpropagation. The preprocessing-normalization cascade and optimized training configuration collectively enabled EfficientNet-B3 to achieve state-of-the-art classification performance while maintaining computational efficiency (20).

The experimental validation confirms that the target detection and classification framework proposed in this study achieves exceptional effectiveness, with particular distinction in its lightweight architecture. Notably, the model operates with a compact parameter size of 15.8M, achieving parameter reductions of 69.04, 86.18, 55.92, and 77.31% relative to the benchmarks set by (34, 42, 44), respectively. This streamlined design substantially reduces computational demands and model complexity while enhancing inference speed, thereby improving operational efficiency and practical deployment viability in real-world scenarios (30).

In target detection tasks, the YOLOv8 model employed in this study demonstrates exceptional performance, achieving an mAP50 of 99.5% and an mAP50–95 of 94.0%. These outstanding metrics conclusively demonstrate the model’s robust capabilities in accurately identifying and localizing anatomical structures (19). For classification, experimental validation was conducted using a clinical dataset of 1,020 X-ray images as the gold-standard validation set. The results revealed an average Top-3 accuracy of 99.04% and a Top-1 accuracy of 85.95% in epiphyseal grade classification, confirming the model’s high precision in this task. Furthermore, the method’s clinical utility is underscored by a remarkably low average absolute bone age estimation error of 0.16 years, solidifying the effectiveness and reliability of the proposed bone age assessment framework (5, 7, 45).

These findings introduce innovative concepts and methodologies to advance bone age assessment research while establishing a robust technical foundation for clinical translation. By incorporating a lightweight architecture, the proposed model not only sets new benchmarks in performance metrics but also achieves substantial improvements in computational efficiency. This dual optimization ensures practical adaptability across diverse healthcare infrastructures, facilitating seamless integration and reliable real-world implementation in medical settings while minimizing operational resource demands (25, 26, 47).

## 5 Conclusions and future research directions

While this study has advanced bone age recognition methodologies, several limitations warrant further refinement. First, in the target detection phase, persistent challenges with incidental inclusion of non-target anatomical regions (e.g., background artifacts) occasionally compromise localization precision. To address this, a methodological refinement could involve integrating a semantic segmentation module prior to detection. Such a module would delineate the precise boundaries of the metacarpal region, thereby eliminating extraneous background elements and ensuring region-specific feature extraction. We hypothesize that this preprocessing step will yield systematic error reduction in detection, improving both robustness and reproducibility of results.

Second, regarding epiphyseal classification, the current methodology predominantly emphasizes isolated feature analysis of individual epiphyses. However, skeletal maturation is a physiological process characterized by coordinated development across multiple growth plates. Sole reliance on single-epiphyseal features risks oversimplification, as it disregards inter-epiphyseal developmental correlations. To mitigate this, future work should adopt a multivariate analysis incorporating developmental correlations among adjacent epiphyseal structures. For instance, leveraging graph-based neural networks to model spatial and developmental dependencies could enable holistic growth pattern recognition. Such an approach, grounded in integration of anatomical prior knowledge, would align computational assessments more closely with clinical interpretations of skeletal maturation.

As deep learning technology advances, increasingly sophisticated object detection and classification models continue to emerge. These innovations provide robust support for the ongoing refinement and enhancement of bone age assessment systems. Moving forward, we aim to harness these cutting-edge advancements in future research to further elevate the accuracy, efficiency, and clinical utility of bone age evaluation. By integrating such technologies, we strive to deliver more precise and reliable diagnostic insights, ultimately strengthening evidence-based decision-making in clinical practice.

## Data Availability

The original contributions presented in the study are included in the article/supplementary material. Further inquiries can be directed to the corresponding author.

## References

[1] Cox LA. The biology of bone maturation and ageing. Acta Paediatr Suppl. (1997) 86:107–8. doi: 10.1111/j.1651-2227.1997.tb18386.x, PMID: 9401555

[2] Cavallo F Mohn A Chiarelli F Giannini C. Evaluation of bone age in children: A mini-Review. Front Pediatr. (2021) 9:580314. doi: 10.3389/fped.2021.580314, PMID: 33777857 PMC7994346

[3] Bass S Pearce G Bradney M Hendrich E Delmas PD Harding A. Exercise before puberty may confer residual benefits in bone density in adulthood: studies in active prepubertal and retired female gymnasts. J Bone Miner Res. (1998) 13:500–7. doi: 10.1359/jbmr.1998.13.3.500, PMID: 9525351

[4] Martin DD Wit JM Hochberg Z Sävendahl L van Rijn RR Fricke O. The use of bone age in clinical practice - part 1. Horm Res Paediatr. (2011) 76:1–9. doi: 10.1159/000329372, PMID: 21691054

[5] Satoh M. Bone age: assessment methods and clinical applications. Clin Pediatr Endocrinol. (2015) 24:143–52. doi: 10.1297/cpe.24.143, PMID: 26568655 PMC4628949

[6] Rösing FW Graw M Marré B Ritz-Timme S Rothschild MA Rötzscher K. Recommendations for the forensic diagnosis of sex and age from skeletons. Homo. (2007) 58:75–89. doi: 10.1016/j.jchb.2005.07.002, PMID: 17306261

[7] Jonvik KL Torstveit MK Sundgot-Borgen J Mathisen TF. Do we need to change the guideline values for determining low bone mineral density in athletes? J Appl Physiol (1985). (2022) 132:1320–2. doi: 10.1152/japplphysiol.00851.2021, PMID: 35060767 PMC9126212

[8] Greulich WW Pyle SI. Radiographic atlas of skeletal development of the hand and wrist. California: Stanford Univ. Press (1959). p. 272.

[9] Cianferotti L Cipriani C Corbetta S Corona G Defeudis G Lania AG. Bone quality in endocrine diseases: determinants and clinical relevance. J Endocrinol Invest. (2023) 46:1283–304. doi: 10.1007/s40618-023-02056-w, PMID: 36918505

[10] Roche AF. Bone growth and maturation. In: Falkner F Tanner JM, editors. Postnatal growth neurobiology. Springer, Boston, MA (1986). p. 25–60. doi: 10.1007/978-1-4899-0522-2_2

[11] Gaskin CM Kahn SL Bertozzi JC Bunch PM. Skeletal development of the hand and wrist: A radiographic atlas and digital bone age companion. New York, USA: Oxford Academic Press (2013). doi: 10.1093/med/9780199782055.001.0001 (Accessed March 25, 2025).

[12] Cameron N. BASIC programs for the assessment of skeletal maturity and the prediction of adult height using the Tanner-Whitehouse method. Ann Hum Biol. (1984) 11:261–4. doi: 10.1080/03014468400007151, PMID: 6547581

[13] Tanner JM Realy J Goldstein H. Assessment of Skeletal Maturity and Prediction of Adult Height (TW3 Method) Vol. 84. . New York. London: Harcourt Publishers (2001) p. 310–1.

[14] Zhang SY Liu LJ Wu ZL Liu G Ma ZG Shen XZ. Standards of TW3 skeletal maturity for Chinese children. Ann Hum Biol. (2008) 35:349–54. doi: 10.1080/03014460801953781, PMID: 18568598

[15] Atwany MZ Sahyoun AH Yaqub M. Deep learning techniques for diabetic retinopathy classification: A survey. IEEE Access. (2022) 10:28642–55. doi: 10.1109/ACCESS.2022.3157632

[16] Amin J Sharif A Gul N Anjum MA Nasir MW Azam F. Integrated design of deep features fusion for localization and classification of skin cancer. Pattern Recognition Lett. (2020) 131:63–70. doi: 10.1016/j.patrec.2019.11.042

[17] Gutierrez L Lim JS Foo LL Ng WY Yip M Lim GYS. Application of artificial intelligence in cataract management: current and future directions. Eye Vis (Lond). (2022) 9:3. doi: 10.1186/s40662-021-00273-z, PMID: 34996524 PMC8739505

[18] Gu Y Chi J Liu J Yang L Zhang B Yu D. A survey of computer-aided diagnosis of lung nodules from CT scans using deep learning. Comput Biol Med. (2021) 137:104806. doi: 10.1016/j.compbiomed.2021.104806, PMID: 34461501

[19] Jocher G Chaurasia A Qiu J. YOLO by ultralytics (2023). Available online at: https://github.com/ultralytics/ultralytics (Accessed January 12, 2025).

[20] Tan M Le Q. (2019). Efficientnet: Rethinking model scaling for convolutional neural networks. Proceedings of the 36th International Conference on Machine Learning, Long Beach, California, PMLR. 97:6105–14.

[21] Liu L Jiang H He P Chen W Liu X Gao J. On the variance of the adaptive learning rate and beyond. arXiv. (2019) 2019. doi: 10.48550/arXiv.1908.03265

[22] Bae C Kim BS. Radiographic study on the time of appearance of the ossification centers in school aged children. J Korean Radiol Soc. (1977) 13:28–34. doi: 10.3348/jkrs.1977.13.1.28

[23] McCarthy SM Ogden JA. Radiology of postnatal skeletal development. Skeletal Radiol. (1982) 7:239–49. doi: 10.1007/BF00361979, PMID: 7071621

[24] Gu G Wu J. Ossification of the hand and wrist in Chinese. Acta Anatom Sin. (1962) 5:173–84.

[25] Li G Zhang D Gao J. Study on bone development in Chinese people I. Preliminary study on bone development of upper limbs. Chin J Radiol. (1964) 9:138–41.

[26] Li G Zhang D Gao J. Study on bone development in Chinese people II. Bone age percentage method. Chin J Radiol. (1979) 13:19–23. 233080

[27] Lee JH Kim KG. Applying deep learning in medical images: The case of bone age estimation. Healthc Inform Res. (2018) 24:86–92. doi: 10.4258/hir.2018.24.1.86, PMID: 29503757 PMC5820091

[28] Hao PY Chokuwa S Xie XH Wu FL Wu J Bai C. Skeletal bone age assessments for young children based on regression convolutional neural networks. Math Biosci Eng. (2019) 16:6454–66. doi: 10.3934/mbe.2019323, PMID: 31698572

[29] Li S Liu B Li S Zhu X Yan Y Zhang D. A deep learning-based computer-aided diagnosis method of X-ray images for bone age assessment. Complex Intell Syst. (2022) 8:1929–39. doi: 10.1007/s40747-021-00376-z, PMID: 34777962 PMC8056376

[30] Li X Jiang Y Liu Y Zhang J Yin S Luo H. RAGCN: Region aggregation graph convolutional network for bone age assessment from X-ray images. IEEE Trans Instrument Meas. (2022) 71:1–12. doi: 10.1109/TIM.2022.3190025

[31] Wang F Gu X Chen S Liu Y Shen Q Pan H. Artificial intelligence system can achieve comparable results to experts for bone age assessment of Chinese children with abnormal growth and development. PeerJ. (2020) 8:e8854. doi: 10.7717/peerj.8854, PMID: 32274267 PMC7127473

[32] Chandran JJG Karthick R Rajagopal R Meenalochini P. Dual-channel capsule generative adversarial network optimized with golden eagle optimization for pediatric bone age assessment from hand X-ray image. Int J Pattern Recog Artif Intell. (2023) 37:2354001. doi: 10.1142/S0218001423540010

[33] Deshmukh S Khaparde A. Faster region-convolutional neural network oriented feature learning with optimal trained recurrent neural network for bone age assessment for pediatrics. Biomed Signal Process Control. (2022) 71:103016. doi: 10.1016/j.bspc.2021.103016

[34] Deshmukh S Khaparde A. Multi-objective segmentation approach for bone age assessment using parameter tuning-based U-net architecture. Multimed Tools Appl. (2022) 81:6755–800. doi: 10.1007/s11042-021-11793-0

[35] Reddy NE Rayan JC Annapragada AV Mahmood NF Scheslinger AE Zhang W. Bone age determination using only the index finger: a novel approach using a convolutional neural network compared with human radiologists. Pediatr Radiol. (2020) 50:516–23. doi: 10.1007/s00247-019-04587-y, PMID: 31863193

[36] Li K Ye K Zhang Z Wang JW Ye LY Zhang QC. Development of hand-wrist bones of 14 year-old adolescents. II. Standard of bony age for girls. Fa Yi Xue Za Zhi. (2008) 24:15–7., PMID: 18404986

[37] Cao L Liu C Wu TH Shi L Wen JX Guo Z. Hand skeletal features of children and adolescents with different growth statuses and periods. Quant Imaging Med Surg. (2024) 14:2528–38. doi: 10.21037/qims-23-26, PMID: 38545069 PMC10963808

[38] Chen XC. Research progress of children’s nutrition in China. Chin J Prev Med. (1999) 33:134–6. 12889464

[39] d’Espaux L Ghosh A Runguphan W Wehrs M Xu F Konzock O. Engineering high-level production of fatty alcohols by Saccharomyces cerevisiae from lignocellulosic feedstocks. Metab Eng. (2017) 42:115–25. doi: 10.1016/j.ymben.2017.06.004, PMID: 28606738

[40] Gilsanz V Chalfant J Kalkwarf H Zemel B Lappe J Oberfield S. Age at onset of puberty predicts bone mass in young adulthood. J Pediatr. (2011) 158:100–105, e1-2. doi: 10.1016/j.jpeds.2010.06.054, PMID: 20797727 PMC4767165

[41] Hägg U Taranger J. Skeletal stages of the hand and wrist as indicators of the pubertal growth spurt. Acta Odontol Scand. (1980) 38:187–200. doi: 10.3109/00016358009004719, PMID: 6932165

[42] Iglovikov VI Rakhlin A Kalinin AA Shvets AA. Paediatric bone age assessment using deep convolutional neural networks. In: Deep learning in Medical Image Analysis and Multimodal Learning for Clinical Decision Support: 4th International Workshop, DLMIA 2018, and 8th International Workshop, ML-CDS 2018, Held in Conjunction with MICCAI 2018, Granada, SPAIN, September 20, 2018, Proceedings, vol. 4. Switzerland: Springer Nature (2018). p. 300–8.

[43] Bui TD Lee JJ Shin J. Incorporated region detection and classification using deep convolutional networks for bone age assessment. Artif Intell Med. (2019) 97:1–8. doi: 10.1016/j.artmed.2019.04.005, PMID: 31202395

[44] Pan X Zhao Y Chen H Wei D Zhao C Wei Z. Fully Automated bone age assessment on large-scale hand X-ray dataset. Int J Biomed Imaging. (2020) 2020:8460493. doi: 10.1155/2020/8460493, PMID: 32190035 PMC7072110

[45] Li Z Chen W Ju Y Chen Y Hou Z Li X. Bone age assessment based on deep neural networks with annotation-free cascaded critical bone region extraction. Front Artif Intell. (2023) 6:1142895. doi: 10.3389/frai.2023.1142895, PMID: 36937708 PMC10017763

[46] Zhang S Shao W Yang JB. Chinese bone maturity evaluation standard and application Vol. 1. Beijing, China: People’s Sports Publishing House (1995). p. 47.

[47] Huang X Wang H She C Feng J Liu X Hu X. Artificial intelligence promotes the diagnosis and screening of diabetic retinopathy. Front Endocrinol (Lausanne). (2022) 13:946915. doi: 10.3389/fendo.2022.946915, PMID: 36246896 PMC9559815

[48] de Margerie-Mellon C Chassagnon G. Artificial intelligence: A critical review of applications for lung nodule and lung cancer. Diagn Interv Imaging. (2023) 104:11–7. doi: 10.1016/j.diii.2022.11.007, PMID: 36513593

